# Neuropathology of the posteroinferior occipitotemporal gyrus in children with autism

**DOI:** 10.1186/2040-2392-5-17

**Published:** 2014-02-24

**Authors:** Neha Uppal, Isabella Gianatiempo, Bridget Wicinski, James Schmeidler, Helmut Heinsen, Christoph Schmitz, Joseph D Buxbaum, Patrick R Hof

**Affiliations:** 1 Fishberg Department of Neuroscience, Icahn School of Medicine at Mount Sinai, One Gustave L Levy Place, Box 1639, New York, NY 10029, USA; 2 Friedman Brain Institute, Icahn School of Medicine at Mount Sinai, New York, NY, USA; 3 Seaver Autism Center for Research and Treatment, Icahn School of Medicine at Mount Sinai, New York, NY, USA; 4 Graduate School of Biomedical Sciences, Icahn School of Medicine at Mount Sinai, New York, NY, USA; 5 Department of Psychiatry, Icahn School of Medicine at Mount Sinai, New York, NY, USA; 6 Department of Natural Sciences, Fordham University, New York, NY, USA; 7 Morphological Brain Research Unit, Department of Psychiatry, University of Wuerzburg, Wuerzburg, Germany; 8 Department of Neuroanatomy, Ludwig-Maximilians University of Munich, Munich, Germany

**Keywords:** Autism, Fusiform gyrus, Neuropathology, Posteroinferior occipitotemporal gyrus, Stereology

## Abstract

**Background:**

While most neuropathologic studies focus on regions involved in behavioral abnormalities in autism, it is also important to identify whether areas that appear functionally normal are devoid of pathologic alterations. In this study we analyzed the posteroinferior occipitotemporal gyrus, an extrastriate area not considered to be affected in autism. This area borders the fusiform gyrus, which is known to exhibit functional and cellular abnormalities in autism.

**Findings:**

No studies have implicated posteroinferior occipitotemporal gyrus dysfunction in autism, leading us to hypothesize that neuropathology would not occur in this area. We indeed observed no significant differences in pyramidal neuron number or size in layers III, V, and VI in seven pairs of autism and controls.

**Conclusions:**

These findings are consistent with the hypothesis that neuropathology is unique to areas involved in stereotypies and social and emotional behaviors, and support the specificity of the localization of pathology in the fusiform gyrus.

## Findings

### Introduction

Neuropathology in autism is typically characterized by subtle alterations in brain regions known to contribute to the behavioral phenotype of the disorder. However, it is important to consider the degree to which these changes are not widespread, but rather are region- and cell-selective, directly influencing the behavior associated with a particular area (for review, see [1]). To assess this further, we analyzed the posteroinferior occipitotemporal gyrus (PIOTG), an extrastriate area directly adjacent to the fusiform gyrus (FG). The FG, which contains the fusiform face area (FFA), is involved in facial processing and therefore is crucial for social communication [2]. As impaired social communication is characteristic of autism [3], a significant effort has been directed towards determining the underlying areas or circuits that may cause this impairment, which often includes the FFA. Many studies report FFA hypoactivation in patients with autism when observing faces (for example, see [4]) and reduced connectivity [5], which may partly explain the social impairments representative of autism. These abnormalities in functional imaging led van Kooten *et al*. [6] to determine whether neuropathologic changes substantiate these functional deficits. In line with their hypothesis, the authors found reduced neuronal number, density, and size in layers III, V, and VI in the FG in patients with autism. No differences were observed in the primary visual cortex, suggesting that while the FG may not properly communicate with areas involved in ‘social’ processing, primary visual information processing is not affected in autism.

Because neuropathologic changes are reported in areas that are both behaviorally and functionally affected, of which the PIOTG is seemingly neither, we did not expect differences in number or size of pyramidal neurons in patients with autism. This result would support the specificity of the neuropathologic data reported by van Kooten *et al*. [6].

## Materials and methods

### Subjects

A total of 12 postmortem brains were analyzed (one hemisphere per case, excluding one autism case and one control for which both hemispheres were available; see Table 1 for details). Accounting for all hemispheres, seven pairs were analyzed. Age range of the cases spanned several developmental stages in order to identify potential developmental changes in the parameters assessed. Tissue processing was performed as previously described [7-9]. This work involved exclusively postmortem materials and was approved by Autism Speaks and the Icahn School of Medicine at Mount Sinai Institutional Review Board. All postmortem materials used in this study were directly obtained from Autism Speaks. All necessary consent was obtained in writing by the patients or their next of kin and confirmed at time of death. Demographic and clinical data are shown in Table 1.

**Table 1:** Demographic and clinical data of patients with autism and controls

**Case**	**Diagnosis**	**Age (years)**	**Sex**	**Hemisphere**	**Cut/mounted thickness (μm)**	**PMI (hours)**	**BW (grams)**	**Cause of death**	**Relevant clinical information**	**ADI-R**
425-02	A1	4	M	L	200/178.5	30	1,160	Drowning	Symptoms present at two years	14, 10 (NV), 3
									Frequent tantrums and self-injurious behavior	
									Used parents’ hand to reach objects, echolalia, tendency to walk on his tiptoes	
									Stereotypic play	
15-763-95	C1	4	M	L	600/574.1	3	1,380	Myocardial infarct	No known disorder	–
425-02	A2	4	M	R	200/165.6	30	1,160	Drowning	Symptoms present at two years	14, 10 (NV), 3
									Frequent tantrums and self-injurious behavior	
									Used parents’ hand to reach objects, echolalia, tendency to walk on his tiptoes	
									Stereotypic play	
15-763-95	C2	4	M	R	600/575.6	3	1,380	Myocardial infarct	No known disorder	**–**
M6-06	A3	7	M	R	200/176.6	25	1,610	Drowning	Regression after first seizure at 14 months	29. 14 (NV), 3
									No spontaneous meaningful language	
									Used parents’ hands as a tool	
									No reciprocal social smiling or eye contact	
									Stereotypic play	
									Psychiatric and developmental disorders on maternal side	
M15-06	C3	7	M	R	200/190.2	12	1,240	Drowning	No known disorder	–
M5-03	A4	8	M	R	200/182.1	22.2	1,570	Rhabdomyo-sarcoma	Symptoms present at three years	19, 14 (V), 4
									Engaged in repetitive play, jumping up and down on his tiptoes, echolalia	
									Cancer diagnosis at age six	
									Abnormal EEG	
M3-04	C4	8	F	R	200/182.8	20	1,222	Multiple injuries	No known disorder	–
427-02	A5	11	F	L	200/177.9	13	1,460	Drowning	Tonic-clonic seizures	22, 14 (V), 3
									Developmental, intellectual, and language delays	
									Poor eye contact	
									Poor social interaction	
M9-03	C5	14	M	R	200/188.8	20	1,464	Electrocution	No known disorder	–
M5-05	A6	17	F	L	200/153.6	25	1,580	Cardiac arrest	Difficulty with reciprocal social interaction	29, 14 (NV), 3
									Appropriate emotional response to her family, but not to others	
									Non-social use of language	
									Stereotypic behaviors	
M16-06	C6	15	F	R	200/174.6	9	1,250	Multiple injuries	No known disorder	–
M1-03	A7	21	F	R	200/263.6	50	1,108	Obstructive pulmonary disease	Seizures	21, 11 (NV), 3
									Engaged in repetitive play, bouncing up and down on her tiptoes, echolalia	
									Abnormal EEG	
M1-07	C7	20	F	R	200/187.1	9	1,340	Multiple injuries	No known disorder	–

ADI-R scores listed as Qualitative Abnormalities in Reciprocal Social Interaction, Qualitative Abnormalities in Communication (V = verbal, NV = nonverbal), and Restricted, Repetitive and Stereotyped Patterns of Behavior, respectively. Cut-off scores for autism are 10, V = 8 or NV = 7, and 3, in order listed above. BW, brain weight; PMI, postmortem interval.

### Regional definition

**Figure 1:**
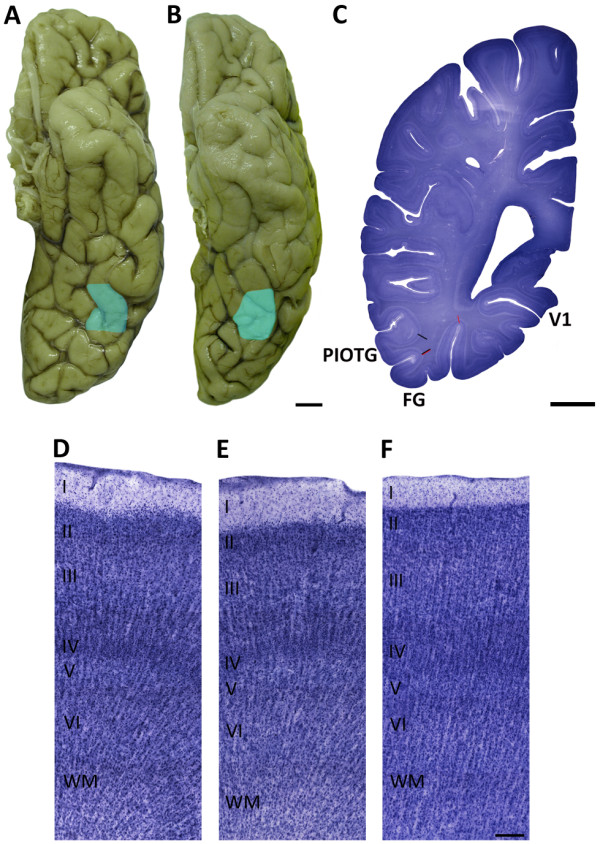
**Cytoarchitectural characteristics of the posteroinferior occipitotemporal gyrus (PIOTG). (A,B)** Left hemisphere of ventral human brain indicating the PIOTG in blue, **(C)** right hemisphere of a coronal human brain section stained with gallocyanin, indicating the PIOTG and FG2 within the two black lines and FG1 within the two red lines, **(D-F)** photomicrographs showing the cytoarchitectural characteristics of (D) the PIOTG, (E) anterior neighboring area 37 and (F) posterior area 19. Scale bar = 2 cm (A, B), 1 cm (C), and 200 μm (D-F). FG, fusiform gyrus; PIOTG, posteroinferior occipitotemporal gyrus; V1, primary visual cortex; cc, corpus callosum; WM, white matter.

Although the visual cortex is rather coarsely parsed into Brodmann areas, the PIOTG has recently been characterized by Caspers *et al*. [10]. The PIOTG (Figure 1A-D) is contained within their area ‘FG2’, lateral to ‘FG1’. FG1 is located on the medial posterior FG, while FG2 is located on the lateral FG, which reaches into the lateral occipitotemporal sulcus [10]. FG2 extends further anteriorly and posteriorly than the expanse of the PIOTG; we restricted our region of interest to accurately and consistently sample the same area in each case.

The PIOTG is a granular cortex, with a dense layer IV and well defined laminar architecture (Figure 1D). Layer II is highly populated, whereas its neighboring layer IIIa is noticeably less dense. Layers IIIb and IIIc have characteristic large pyramidal neurons, and the density of neurons increases near IIIc. The border between layer IIIc and layer IV is somewhat indistinct, though layer IV is distinguishable by its very dense and granular appearance. The medium-sized pyramidal neurons in layer V are evenly concentrated throughout the layer, contrasting with the denser layer VI.

The PIOTG was defined anteriorly by the caudal-most level of the subiculum, and posteriorly by marked columnar neuronal organization. Thus, the PIOTG spans part of Brodmann area 37 (Figure 1E) and extends partially into area 19 (Figure 1 F; [11]). The cytoarchitecture of this junctional region was clearly described by von Economo [12], and we adopted his definition for this analysis. The temporo-occipital junction has a clear lamination pattern in layer III and a thin radial striation at times perceptible into layer IV. Layer V contains small pyramidal cells, with an indistinct border with layer VI. Extending posteriorly into area 19 is a progressively denser layer II, an increased cellularity of layer III, and isolated large neurons close to a distinct and dense layer IV. Layer V is rather thin with small pyramidal neurons and isolated large neurons in its upper border, with a denser layer VI. We relied on the detailed description of Caspers *et al*. [10] for the dorso-ventral boundaries of our region of interest, within the more global context of the classical descriptions by Brodmann and von Economo [11,12].

### Stereologic design

For stereologic quantification, we selected every available section within the range of sections containing the PIOTG for each case (1:3 series for 200-μm sections, 1:2 for 500- and 600-μm sections). The mounted thickness of these materials was calculated by averaging the measurements of a random sampling of ten sites per slide (40× objective) in the PIOTG.

Sampling grid dimensions were set to sample pyramidal neurons such that the coefficient of error was ≤ 0.1 [13]. StereoInvestigator (MBF Bioscience, Willinston, VT, USA) defined a systematic-random sampling sequence of counting frames and grids within the outlines of each layer analyzed in the PIOTG, in which pyramidal neurons were quantified. Layers were defined at 2.5× and quantification occurred at 40×. Neurons were counted according to stereologic principles, estimating cell population with the Optical Fractionator [14], pyramidal neuron volume with the Nucleator [15], and total layer volume with the Cavalieri principle ([13,16]; Table 2).

**Table 2:** Stereologic parameters used for pyramidal neuron quantification in control subjects and patients with autism

	
Number of sections (average)	7.7
Objective 1	2.5×
Objective 2	40×
Disector height	20 μm
Guard zone	5 μm
Counting frame	75 × 75 μm
Grid size	Layer III: 750 × 750 μm
	Layer V: 500 × 500 μm
	Layer VI: 500 × 500 μm
Measured thickness (μm, average)	Cut at 200 μm: 185.1
	Cut at 600 μm: 574.8
Nucleator rays	4
Cavalieri grid size	100 × 100 μm

### Statistical analysis

We compared seven hemispheres of patients with autism to seven hemispheres of controls. For statistical analysis, we used a matched-subject design, comparing each patient with an age-matched control using a paired *t*-test. Because pairs 1 and 2 compared two hemispheres from the same patient and control, we also conducted paired *t-*tests excluding either pair 1 or 2 to determine whether having both hemispheres confounded the results. Additionally, we used repeated measures analysis of variance to control for potential effects of age, gender, and postmortem interval. A *P*-value of 0.05 was used as the criterion for statistical significance. Calculations were performed with GraphPad Prism (version 5.03, GraphPad Software, San Diego, CA, USA) and SPSS (version 20, SPSS Inc., Chicago, IL, USA).

## Results

Upon visual inspection, patients with autism and controls showed a similar cytoarchitecture, characteristic of the PIOTG (Figure 1D). We assessed pyramidal neuron number, pyramidal neuron volume, and layer size in layers III, V, and VI in patients with autism and control subjects.

**Figure 2:**
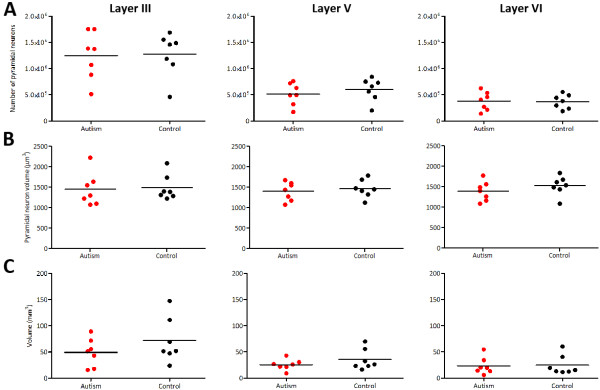
**Graphs depicting quantitative parameters measured in patients with autism and controls.** Typically developing controls are shown in black and patients with autism in red. Note the lack of differences in estimated neuron population **(A)**, neuron size **(B)**, and layer volume **(C)** between control cases and patients with autism in layers III (left column), V (middle column), and VI (right column).

**Figure 3:**
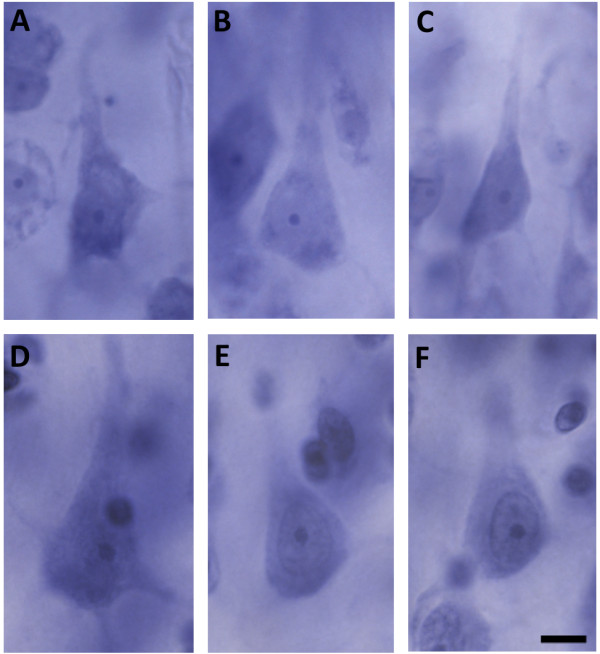
**Pyramidal neuron volume is not visibly different in patients with autism and controls.** Photographs of pyramidal neurons in patients with autism in layer III **(A)**, layer V **(B)**, and layer VI **(C)**, compared to pyramidal neurons in control subjects in layer III **(D)**, layer V **(E)**, and layer VI **(F)** in the posteroinferior occipitotemporal gyrus (PIOTG). Scale bar = 10 μm.

The paired samples *t*-test resulted in no significant differences in pyramidal neuron number in layers III (*t*_(6)_ = 0.091, *P* = 0.9306), V (*t*_(6)_ = 0.709, *P* = 0.5047), and VI (*t*_(6)_ = 0.091, *P* = 0.9305; Figure 2A). There was also no statistically significant difference in pyramidal neuron volume in layers III (*t*_(6)_ = 0.038, *P* = 0.7169), V (*t*_(6)_ = 1.023, *P* = 0.3457), and VI (*t*_(6)_ = 1.32, *P* = 0.2349; Figure 2B, Figure 3). Volume of layers III (*t*_(6)_ = 1.236, *P* = 0.2625), V (*t*_(6)_ = 1.1, *P* = 0.3136), and VI (*t*_(6)_ = 0.141, *P* = 0.8928) were also not statistically different in patients with autism and controls (Figure 2C). Additionally, pyramidal neuron density was not statistically different in layers III (*t*_(6)_ = 1.4, *P* = 0.211), V (*t*_(6)_ = 0.271, *P* = 0.7952), and VI (*t*_(6)_ = 0.278, *P* = 0.7905; Table 3). These results were still non-significant when either pair 1 or 2 was excluded from analysis (Table 4). In addition, when each covariate (age, gender, and postmortem interval) was added using repeated measured analysis of variance, we did not find any statistically significant differences between patients with autism and controls in any of the parameters assessed.

**Table 3:** Summary of stereologic results in control subjects and patients with autism

**Variable**	**Controls (mean ± SD)**	**Autism (mean ± SD)**
Pyramidal neuron number		
Layer III	1.274 × 10^6^ ± 415515	1.247 × 10^6^ ± 456858
Layer V	600641 ± 217605	512054 ± 213975
Layer VI	369762 ± 135770	378581 ± 176463
Pyramidal neuron volume (μm^3^)		
Layer III	1491 ± 309.1	1446 ± 403.2
Layer V	1468 ± 220.0	1400 ± 225.7
Layer VI	1527 ± 234.2	1395 ± 239.2
Layer volume (mm^3^)		
Layer III	49.26 ± 26.66	71.77 ± 42.69
Layer V	25.52 ± 10.28	35.27 ± 19.79
Layer VI	23.23 ± 16.4	24.71 ± 18.6
Pyramidal neuron density (1/mm^3^)		
Layer III	32687 ± 24425	20882 ± 8094
Layer V	20738 ± 6849	19826 ± 9099
Layer VI	19673 ± 8341	18304 ± 6800

**Table 4:** Summary of statistical results in control subjects and patients with autism

**Variable**	***P*-value for all pairs**	***P*-values without pair 1**	***P*-values without pair 2**
Pyramidal neuron number			
Layer III	0.9306	0.9736	0.858
Layer V	0.5047	0.6099	0.599
Layer VI	0.9305	0.6718	0.8253
Pyramidal neuron volume			
Layer III	0.7169	0.7121	0.5754
Layer V	0.3457	0.328	0.2466
Layer VI	0.2349	0.3165	0.1626
Layer volume			
Layer III	0.2625	0.5003	0.4574
Layer V	0.3136	0.6044	0.5659
Layer VI	0.8928	0.6253	0.8258
Pyramidal neuron density			
Layer III	0.2110	0.2596	0.3386
Layer V	0.7952	0.9194	0.4404
Layer VI	0.7905	0.9106	0.7668

## Discussion

The purpose of this study was to identify whether an area with little-known relevance to autism, the PIOTG, exhibited neuropathologic features present in areas associated with the behavioral abnormalities of autism. The significance of the PIOTG in particular is its proximity to the FG, an area well documented to be functionally and anatomically altered in autism. In line with our hypothesis, we found no significant differences in pyramidal neuron number, pyramidal neuron size, pyramidal neuron density, or layer volume in patients with autism compared to age-matched controls (Figures 2, 3). It is important to take the small sample size into consideration, as this is a limitation and does not allow for a formal power analysis; a larger cohort of cases would be more sensitive to potential neuropathology. In addition, it is of note that as demonstrated by the PIOTG, a longer postmortem interval and drowning as a cause of death are two factors that do not result in cytoarchitectural abnormalities in the measured parameters.

Although it is difficult to draw conclusions on functional correlates to an area that has only been characterized morphologically, Caspers *et al*. [10] suggested that FG2 is within a cortical region involved in higher-order object-related processing. More specifically, FG2 may correlate to a ‘patch’ of the FFA. Although this potential participation in face processing suggests an involvement in autism behaviorally, the role of the PIOTG is likely more general, based on its anatomical location (face processing becomes progressively more complex in more anterior areas). In fact, Caspers *et al*. confirmed the role of FG2 in face processing, and also uncovered functional lateralization in FG2: the left FG2 is active during visual language processing, while the right FG2 is more active during face processing [17]. FG2 participates in several functions related to visual and face processing, but the principal activation patterns suggest that it is situated early in the functional hierarchy of face processing, and is likely involved in pattern analysis [17]. The neuropathology in face processing areas occurs in areas predominantly involved in emotional or affective characteristics of faces, which FG2 (and therefore the PIOTG) are not. That being said, the PIOTG is still part of the face processing pathway, albeit early on, and there is a possibility that it is affected indirectly. Although all analyzed parameters did not reveal significant differences, patients with autism showed a slight trend towards a reduction for some of the assessed variables, as seen visually in Figure 2. This may indicate a peripheral effect from areas that interact with the PIOTG (for example, the FG, in which patients with autism have neuropathology [6]).

Overall, the PIOTG shows involvement in attention-demanding visual processing, primarily in visual object recognition [10,14]. This higher-order visual area shows hemispheric lateralization that suggests functions in language, word, and face processing. Although we were unable to parse out potential hemispheric differences, we confirmed a lack of neuropathology in the PIOTG. This result is consistent with the concept that areas uninvolved in social, emotional, and stereotypic behaviors would not show cellular pathology in autism. This study provides a foundation for continuing efforts to characterize the brain in autism, as area-specific neuropathology can provide insight into potential altered cortical functioning.

## Availability of supporting data

The data sets supporting the results of this article are included within the article; raw data are shared on the Autism Speaks portal.

## Abbreviations

FFA: fusiform face area; FG: fusiform gyrus; PIOTG: posteroinferior occipitotemporal gyrus.

## Competing interests

The authors declare that they have no competing interests.

## Authors’ contributions

NU: conception and design, data collection, analysis, and interpretation, manuscript preparation, and final approval of the manuscript. IG: data collection, manuscript preparation, and final approval of the manuscript. BW: data collection, manuscript preparation, and final approval of the manuscript. JS: data analysis and interpretation, critical revision and final approval of the manuscript. HH: processed all materials, critical revision and final approval of the manuscript. CS: conception and design, processed all materials, data interpretation, critical revision, and final approval of the manuscript. JDB: conception and design, data interpretation, critical revision and final approval of the manuscript. PRH: conception and design, data interpretation, manuscript preparation, and final approval of the manuscript. All authors read and approved the final manuscript.
